# Smart home energy management and power trading optimization using an enhanced manta ray foraging optimization

**DOI:** 10.1038/s41598-023-49176-0

**Published:** 2023-12-13

**Authors:** Heba Youssef, Salah Kamel, Mohamed H. Hassan

**Affiliations:** 1 Department of Electrical Engineering, Faculty of Engineering, Aswan University, Aswân, 81542 Egypt; 2 Ministry of Electricity and Renewable Energy, Cairo, Egypt

**Keywords:** Computational science, Software, Energy infrastructure

## Abstract

This paper proposes a plan to manage energy consumption in residential areas using the demand response method, which allows electricity users to contribute to the reliability of the power system by controlling their usage. Due to the growing population, the residential sector consumes a significant amount of energy, and the objectives of this study are to lower electricity costs and the peak to average ratio, as well as reduce the amount of imported electricity from the grid. The study aims to maximize profit by properly utilizing renewable energy sources and addressing energy trading. The manta ray foraging optimization (MRFO) and long term memory MRFO (LMMRFO) algorithms are used to solve this problem. Firstly, the validation of the proposed LMMRFO technique is confirmed by seven benchmark functions and compared its results with the results of the well-known optimization algorithms including hunter prey optimization, gorilla troops optimizer, beluga whale optimization, and the original MRFO algorithm. Then, the performance of the LMMRFO is checked on the optimization of smart home energy management. In the suggested approach, a smart home decides whether to purchase or sell electricity from the commercial grid based on the cost, demand, and production of electricity from its own microgrid, which consists of a wind turbine and solar panels. Energy storage systems support the stable and dependable functioning of the power system since the solar panel and wind turbine only occasionally produce electricity. Through various case studies, the proposed plan is tested and found to be effective in reducing electricity costs and the peak to average ratio while maximizing profit. Furthermore, a comparative study is conducted to demonstrate the legality and effectiveness of LMMRFO and MRFO.

## Introduction

The increasing global population, climate change, escalating carbon emissions, and surging electricity demand have placed electricity production and distribution entities, as well as governments, in a challenging situation where decisive actions to tackle these critical issues are impeded. Power-producing enterprises find it difficult to embrace renewable energy sources (RESs) as a solution to combat global warming and reduce carbon emissions^1^. The existing electrical network provides a significant number of supply lines with low voltage load for residential customers and small businesses. Due to the centralized approach, the power flow in the current power system is unidirectional. Consumers of electricity are only passive users and have no effect on the dependability and stability of the electrical grid^2^. The current power system's a lack of monitoring technology and unidirectional communication are the main causes of electricity waste. The innovative strategy is spread based on two-way communication. It offers a more widespread and distributed intelligence in the production, transmission, and use of electricity. Additionally, the innovative strategy offers power users a variety of ways to control their usage for lower bills and dependable grid operation. To address the aforementioned issues in the conventional electric grid, the solution is to integrate renewable energy sources with the smart grid. Electricity is produced in the smart grid using more affordable and effective resources, and it is then transmitted to users via smart transmission lines. The term "electricity users" refers to people who can use and generate electricity from their own local microgrid. It is made up of several RESs, such as hydro power plants, wind turbines, solar panels, etc.

The users connect to the industrial grid and use electricity that they produce themselves. They buy electricity from utilities if there is less electricity generation than load demand. If their own microgrid produces more electricity than is needed to meet the load, the extra energy is stored in batteries for use at a later time when electricity generation is low. Only when the microgrid's electricity production is low or the cost of electricity per unit is high are the batteries allowed to drain. For the purpose of motivating users to minimize their electricity usage during ON-peak times, there exist numerous dynamic pricing schemes. Critical peak rebate (CPR), critical peak pricing (CPP), time of usage (ToU), and real-time pricing (RTP) are a few of these pricing models. Customers that use electricity can choose the best electricity rate based on their preferences^3^.

Currently, RESs produce just a few MW or kW of electricity in residential areas, while large-scale RES integration is widely dispersed throughout the world. Furthermore, smart homes and small businesses can make money by reselling surplus electricity to nearby neighbors or the grid thanks to RES-based electricity generation and storage system integration. Additionally, end users can buy energy while power rates are low and trade it back if costs are high. Additionally, demand side management (DSM) can reduce electricity use by between 10 and 30 percent^4^.

The reductions of PAR and consumption cost optimization are both possible in smart grids thanks to two-way communication. Numerous studies have concentrated on the cost and PAR reduction provided by DSM as a result of the development of a smart grid^5–7^. However, the entirety of this research has not encompassed the inclusion of electricity production and storage for subsequent usage. The smart home with several smart device kinds^8^, the optimal electricity usage with maximum consumer comfort is established. They also look at their suggested smart construction plan, which consists of several smart dwellings with various living styles such as load demand and power rating. Ahmad et al.^9^ introduced a DSM model by taking into account various power customers. For power users, the authors of^10^ have suggested a brand-new smart grid architecture. For the generation of electricity, they also incorporate RESs. Their research indicates that the consumers have the ability to produce, use, store, and sell extra electricity. In order to maximize profits, the extra electricity is sold back to the commercial grid. The latest developments suggested solutions consider the issue from the grid's or electrical users' perspectives. To cut down on electricity costs and carbon emissions, the authors of^11^ also incorporate RESs.

In this paper, a DSM scheme for combining energy storage systems (ESS) and RESs in a residential area has been presented to reduce PAR and power costs. We take into account a smart house that can produce and use electricity from its own mini-grid and store the excess for later use as well as use electricity from the public grid. The smart house is also liberated from taking decisions on its own for reducing electricity costs and PAR while maximizing earnings for every hour. Consequently, increasing the funds from power exchanging is another goal of this study. The smart house takes decisions based on the cost of electricity. The residence makes an effort to minimize load demand when prices are high, and any extra electricity is resold to the industrial grid. The residence minimizes the PAR by buying electricity during periods of lower cost. For the purpose of calculating the cost of selling and buying power, two separate RTP schemes are also taken into account. A comparison of the LMMRFO and MRFO algorithm performance is done in the conclusion. The main contributions of this paper are as follows:

- Utilizing the LMMRFO and MRFO heuristics to efficiently schedule devices for power trading, enabling optimal energy management in the smart home.
- Application of the optimization algorithm to a real-world problem in home energy management, leading to practical implications such as optimized power consumption, cost reduction, and minimization of the peak to average ratio (PAR).
- Integration of renewable energy sources (RESs), such as wind turbines and solar panels, into the optimization framework, demonstrating the algorithm's practical applicability in green energy systems.
- Development of a system that empowers smart homes to autonomously make decisions regarding energy consumption and trading with the grid based on electricity prices. This promotes energy efficiency and cost savings.
- Performance evaluation of the algorithm through comprehensive simulations considering various metrics, including total cost, PAR, and earnings. This evaluation provides valuable insights into the effectiveness and practical benefits of the proposed approach.
- Conducting comprehensive simulations using MATLAB (2018a) to validate the effectiveness of LMMRFO and MRFO.

The rest of the paper is structured as follows: In section "Related work", an overview of the literature is given. The problem description and suggested system model are described in section "Model of proposed system". In section "Proposed Algorithm", we describe our suggested plan. section "Benchmark functions" presents the simulation results using the benchmark functions. section "Scenario Studies" provides an explanation of case studies, and section "The Results" provides the findings of the simulation. In section "Conclusions", a paper finding is explained.

## Related work

In the past few decades, academics have focused on two important and difficult research problems: lowering the cost of electricity and achieving load equilibria between supply and demand. In the recent years, a variety of DSM techniques have been proposed to minimize electricity costs and PAR while maximizing user satisfaction. Below is a presentation of some of the current research.

In^12^, an approach based on integer linear programming (ILP) is suggested. Finding a balance among power supply and demand in the housing locality is the main goal of this study. For time- and power-shiftable devices, their suggested technique effectively shifts the optimal operating time and power. According to experimental findings, the technique they suggested effectively archived the stated goals. An mixed integer linear programming (MILP)-based load balancing strategy and cost reduction in a residential neighborhood is shown in^11^. Electricity users are regarded as consumers by a MILP-based HEMS^10^. For an individual smart house and a group of 39 prosumers, they presented HEMS. Each smart home has a set of solar panels on it to generate electricity, and it is additionally linked to a public grid to help it fulfill request. When their generation exceeds their needs, consumers store excess energy in batteries. To maximize profits, they are unable to export electricity during times when it is most expensive. In^13^, a mixed integer linear programming (MILP)-based scheduling method for household appliances was put forth to lower overall electricity costs and balance load demand in residential settings. The experimental findings show that their suggested strategy quickly meets the required goals, namely the decrease of peak load and power cost. The authors of 13 presents a plan for minimizing costs by incorporating dispersed energy sources. Additionally, each user has a solar panel and battery system for producing electricity. Customers of electricity have the option to sell or buy it based on price indications. In their work, the cost of electricity is calculated using an RTP system. However, because there are devices that can be interrupted, like TVs, etc., this study only takes non-interruptible equipment into account, which is unrealistic for a smart house. Zhang et al.^14^ presented a MILP-based methodology for reducing electricity costs and PAR as well as integrating RESs. The simulation findings support the suggested model for reducing PAR and power costs with effective RES integration. GA and Particle swarm optimization (PSO) were also suggested as a heuristic-based technique by Khan et al.^15^. Three alternative pricing signals ToU, RTP, and CPP along with a knapsack problem are applied to design the optimization problem. The major goals of this endeavor are to reduce peak demand and electricity costs. Experimental investigation demonstrates the effectiveness of the suggested PSO and GA method. Additionally, they compare GA to PSO, demonstrating that GA performs better than PSO. The authors of^16^ used a genetic algorithm to make balance load between electricity demand and supply and reduce energy cost. The authors in^17^ used A dynamic programming (DP) to reduce electricity costs. Home appliances can be scheduled to run during off-peak hours to save money on electricity. Additionally, a game theory-based strategy is used to engage power users while generating additional electricity. Agnetis et al.^18^ solved three the problem of optimization, which are cost minimization, maximization of user comfort and scheduling preference optimization. The problem of electricity bill optimization in a ToU system is described in^19^. Additionally, they divide the overall load into 3 groups: shiftable load, weather-based load, and interruptible load. In^20^, a GA-based plan for reducing the cost of power with integrated ESS and RES was given. The energy storage system is applied to equilibrium the demand and supply for electricity. An empirical investigation demonstrates the effectiveness of their suggested plan. According to^21^, the HEMS use mixed integer linear programming to modify the load request between the electric vehicles and ESS, electric grid and PV panels. The DR policy, which is described in^22^, aims to save money on electricity and reduce PAR by scheduling smart devices in accordance with hourly electricity costs.

The integration of RESs into an intelligent HEMS was presented in^23^ for the reduction of power costs and PAR. The trade issue in smart grids was examined by the authors of^24^. Users of electricity have the option to generate, buy, and sell electricity. However, rather than selling to a single utility, they put their surplus of electricity up for auction. The capability of energy users to concurrently produce, retain, and alter their usage in accordance with electricity tariffs, as well as sell extra electricity back to the electric grid, hasn't been taken into account in^10,11,13,24^. Here, we provide a brand-new energy management strategy that uses LMMRFO and MRFO to address the issues outlined above.

The author of reference^25^ presents a thorough examination of efficient HEMS within the context of modern smart grids and advancing technologies. The review encompasses technical aspects, conceptual frameworks, and challenges associated with HEMS. Additionally, it introduces an innovative approach that incorporates green building concepts into home design, aiming to decrease energy consumption. The paper highlights the significance of not only developing energy-efficient models and appliances but also promoting user awareness and active participation in energy conservation. The study showcases the effects of different strategies on reducing peak loads, with the Optimization-based Residential Energy Management technique achieving a substantial 35% decrease in overall electricity bills.

In reference^26^, the paper focuses on utilizing the Sine Cosine Algorithm to optimize HEMS, with a particular emphasis on load shifting through Demand Side Management (DSM) in smart homes. The primary goal of this optimization is to reduce electricity bills and Peak-to-Average Ratio (PAR) while maintaining consumer comfort. The study takes into account various pricing signals and demonstrates significant reductions in electricity bills (up to 40%) and PAR (up to 50%) without significant impacts on electricity costs and PAR when devices are coordinated in real-time.

This research employs the Improved Bald Eagle Search Optimization Algorithm to create an efficient HEMS for smart homes^27^. Its primary objectives are to handle load demand, optimize energy usage, reduce electricity expenses, decrease average peak ratios, and improve user comfort. To achieve these goals, a load conversion strategy is implemented to effectively coordinate household appliances, minimizing peak-to-average ratio (PAR) and electricity costs. Real-time scheduling of daily activities and dynamic programming are employed to address rescheduling challenges. The study assesses the algorithm's performance under various pricing strategies, improved user convenience, reduced peak ratios, and cost savings.

The author of^28^ concentrates on improving the energy efficiency of buildings to meet economic and environmental goals. They utilize an elite evolutionary strategy in an artificial ecosystem optimization approach to optimize the scheduling of electrical appliances in smart homes, incorporating load conversion as a demand-side management (DSM) strategy. It's worth noting that this paper conducts a comparative analysis between the proposed algorithm and the original one to validate its effectiveness.

## Model of proposed system

**Figure 1:**
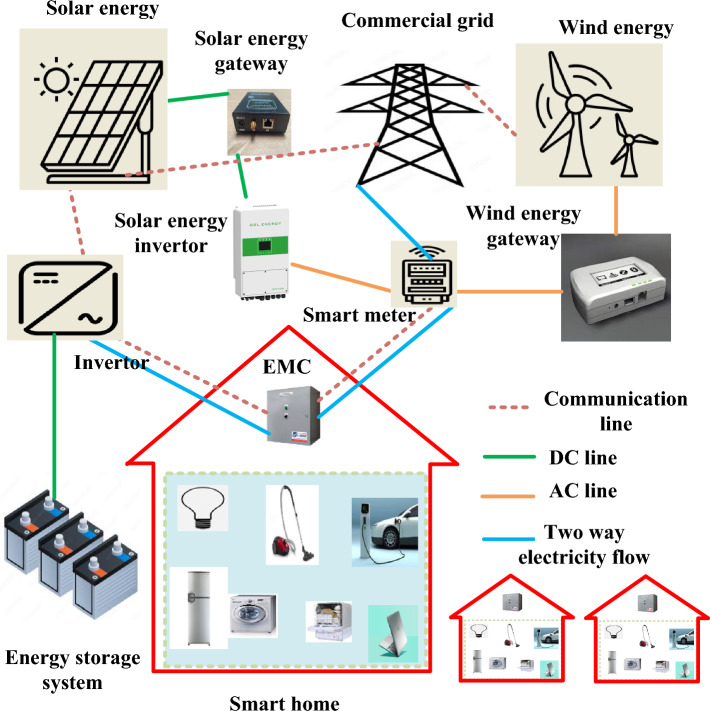
system model.

The goal of this paper is to develop a design for future smart grids that will reduce power costs for residential users, stabilize the system, and reduce peak load across the board. Several smart devices with varying operational times and power ratings are included in the smart home. The solar panel and wind turbine make up the microgrid. Due to the intermittent nature of these distributed EGSs, ESS, or batteries, are also installed to meet the load requirements of users. The shortfall load is brought from the ESS or utility, and electricity costumers generate all of their own microgrid to fulfill their own energy needs. Consumers of electricity occasionally have extra energy leftover after use. In this case, electricity users have the option of selling their excess electricity to the industrial grid. The suggested optimization model is described below and shown in Fig. 1. ^29^ Additionally, we take into account an electric system with a single utility provider that has smart grid features includes effective monitoring, bidirectional communication, etc. The numerous consumers are all supplied with energy by the same utility company. Each consumer has their own distributable energy generating system (EGS), or microgrid, as well as numerous smart devices that use electricity.

### Objective functions and constraints

The primary focus of our study is to minimize the total energy cost for the smart home while reducing the PAR of electricity consumption and maximizing earnings through power trading. This study presents an improved algorithm that surpasses the performance of the original one. Furthermore, we've incorporated a benchmark section to demonstrate the remarkable enhancements achieved by the enhanced algorithm compared to the original. The enhanced algorithm makes a substantial contribution to the effective scheduling of devices for power trading, facilitating optimal energy management within a smart home.

Utilizing the improved algorithm to address real-world challenges within home energy management, resulting in tangible benefits like optimized power consumption, cost reduction, and PAR minimization. Incorporating RESs, such as wind turbines and solar panels, into the optimization framework, showcasing the practical application of the algorithm in green energy systems. Creating a system that empowers smart homes to autonomously make decisions concerning energy consumption and grid-based trading, guided by electricity prices. This fosters energy efficiency and financial savings.

#### Home electricity load

In this study, we take a 24-h implementation into account, with 1 h allotted for each time slot written. The single time slot is represented by $t$ and the sum of the daily time slots by T. The household also includes a variety of devices, which are further divided into three primary groups: base-load devices (${D}_{b}$), shiftable devices (${D}_{s}$), and non-interruptible devices (${D}_{ni}$). ${D}_{n}$ is the total number of devices. Each device also has an internet connection and can communicate with the energy management controller (EMC). EMC has a Wi-Fi connection to the internet, which enables it to adjust appliance operation to our fitness needs. Every smart device must finish its operational time in our system model. Table 1 shows the parameters for devices. The values for the device parameters mentioned in Table 1 were obtained from^29^. Additionally, the classification of household devices is described in the following part.

**Table 1:** Device parameters for the home.

Device category	Device name	Least finishing time (h)	Earliest starting time (h)	Varying operational times (h)	Power rating (kw)
Base load	Interior lighting	24	16	6	0.84
	Refrigerator	24	1	24	0.3
Non-interruptible	Dish washer	17	9	2	1.5
	Washing machine	15	7	2	1.5
	Spin dryer	18	13	1	2.5
Shiftable	Cooker hub	10	6	1	3
	Cooker oven	20	15	1	5
	Microwave	10	6	1	1.7
	Laptop	24	18	2	0.1
	Desktop	24	18	3	0.3
	Vacuum cleaner	17	9	1	1.2
	Electrical car	8	18	3	3.5

##### Base devices

The base devices ${D}_{B}$ are the kind of devices that can't be moved or stopped in the middle of a task. These devices, also known as non-interruptible and non-shiftable devices, are typically thought of as the primary load in each household. We classify refrigerators and interior lighting as base devices. Each device in this category is represented by a power rating (${\lambda }_{b}$), and the total amount of electricity (${E}_{b}$) used in a day is computed as follows:

1 $${E}_{b}=\sum_{t=1}^{T} \left(\sum_{{d}_{b}\in {D}_{b}} \left({\lambda }_{b}\times {\alpha }_{b}(t)\right)\right)$$

Equations (2) and (3) are used, respectively, to determine the hourly and daily cost against utilized electricity:

2 $${\sigma }_{{d}_{b}}^{t}=\sum_{{d}_{b} \in { D}_{b}} \left({\lambda }_{b}\times \rho (t)\times {\alpha }_{b}(t)\right)$$

3 $${\delta }_{{d}_{b}}^{\mathrm{Total }}=\sum_{t=1}^{T} \left(\sum_{{d}_{b}\in { D}_{b}} \left({\lambda }_{b}\times \rho (t)\times {\alpha }_{b}(t)\right)\right)$$

${\alpha }_{b}(t)$ represents the OFF/ON status of base device:

4 $${\alpha }_{b}(t)=\left\{\begin{array}{ll}1,& \mathrm{ If }\;{d}_{b}\;\mathrm{ is }\;ON\\ 0,& \mathrm{ If }\;{d}_{b}\;\mathrm{ is }OFF\end{array}\right.$$

##### Non-interruptible devices

The second group of devices, known as non-interruptible devices, is defined in this section. This kind of device may not be stopped once execution has begun, but may be moved to another time slot before it does. Non-interruptible devices cannot have their operation time modified. This kind of device can be scheduled between the earliest starting and the latest finishing times. Let ${d}_{ni }\in {D}_{ni}$ stand in for each device under this heading. Each device in this category is represented by a power rating (${\lambda }_{ni}$), and the total amount of electricity (${E}_{ni}$) used in a day is computed as follows:

5 $${E}_{ni}=\sum_{t=1}^{T} \left(\sum_{{d}_{ni}\in {D}_{ni}} \left({\lambda }_{ni}\times {\alpha }_{ni}(t)\right)\right)$$

Equations (6) and (7) are used, respectively, to determine the hourly and daily cost against utilized electricity:

6 $${\sigma }_{{d}_{ni}}^{t}=\sum_{{d}_{ni }\in {D}_{ni}} \left({\lambda }_{ni}\times \rho (t)\times {\alpha }_{ni}(t)\right)$$

7 $${\delta }_{{d}_{ni}}^{\mathrm{Total }}=\sum_{t=1}^{T} \left(\sum_{{d}_{ni}\in {D}_{ni}} \left({\lambda }_{ni}\times \rho (t)\times {\alpha }_{ni}(t)\right)\right)$$

${\alpha }_{ni}(t)$ represents the OFF/ON status of base device:

8 $${\alpha }_{ni}(t)=\left\{\begin{array}{ll}1,& \mathrm{ If }\;{d}_{ni}\;\mathrm{ is }\;ON\\ 0,& \mathrm{ If }\;{d}_{ni}\;\mathrm{ is}\; \mathrm{OFF}\end{array}\right.$$

##### Shiftable devices

The third group of devices, known as shiftable devices, is defined in this section. Depending on their use, various types of devices may be moved or stopped at any time. The class of movable devices includes laptops, vacuum cleaners, electric vehicles, etc. Each device in this category is represented by a power rating (${\lambda }_{s}$), and the total amount of electricity (${E}_{s}$) used in a day is computed as follows:

9 $${E}_{\mathrm{s}}=\sum_{t=1}^{T} \left(\sum_{{d}_{s} \in {D}_{s}} {\lambda }_{s}\times {\alpha }_{\mathrm{s}}(t)\right)$$

Equations (10) and (11) are used, respectively, to determine the hourly and daily cost against utilized electricity:

10 $${\sigma }_{{D}_{s}}^{t}=\sum_{{d}_{s} \in { D}_{s}} \left({\lambda }_{s}\times \rho (t)\times {\alpha }_{\mathrm{s}}(t)\right)$$

11 $${\delta }_{{D}_{\mathrm{s}}}^{\mathrm{Total }}=\sum_{t=1}^{T} \left(\sum_{{d}_{s} \in { D}_{s}} \left({\lambda }_{s}\times \rho (t)\times {\alpha }_{\mathrm{s}}(t)\right)\right)$$

${\alpha }_{s}(t)$ represents the OFF/ON status of base device:

12 $${\alpha }_{s}(t)=\left\{\begin{array}{ll}1,& \mathrm{ If }\;{d}_{s}\;\mathrm{ is }\;ON\\ 0,& \mathrm{ If }\;{d}_{s}\;\mathrm{ is }\;OFF\end{array}\right.$$

#### Microgrid

We take into account the neighborhood microgrid with various renewable energy sources, such as wind turbines and solar panels. Equations (13) and (14), respectively, are used to express how much electricity the microgrid generated during the time period $t \in T$ and during the entire day:

13 $$E(T)=\sum_{m\in M} {\varepsilon }_{m}(t)$$

14 $$E=\sum_{t}^{T} \sum_{m\in M} {\varepsilon }_{m}(t)$$

The single time slot $t$ and maximum time slot $T$ are denoted in Eqs. (13) and (14), respectively. The fact that RESs are intermittent in nature is one thing to keep in mind^30^. There are numerous statistical models available to forecast future RES electricity generation.

#### Wind turbine

A wind turbine uses the kinetic energy to create electricity. Equation (15) explains how the wind turbine ${P}_{t}^{wt}$ in time $t$ generates electric power. The area of wind turbine blades, the speed of wind, and efficiency of wind turbines are used in this equation to compute the electric energy. Between the wind's cut-out and cut-in speeds, wind turbine produces electric energy. Equations (16) – (18) present all restrictions for wind turbines.

15 $${\mathrm{P}}_{\mathrm{t}}^{\mathrm{wt}}=1/2\cdot {\mathrm{C}}_{\mathrm{p}}\cdot (\uplambda )\cdot\uprho \cdot \mathrm{A}\cdot {\left({\mathrm{V}}_{\mathrm{t}}^{\mathrm{wt}}\right)}^{3}$$

16 $${\mathrm{V}}^{\mathrm{cut}-\mathrm{in}}\le {\mathrm{V}}_{\mathrm{t}}^{\text{wt }}\le {\mathrm{V}}^{\mathrm{cut}-\mathrm{out}},\forall \mathrm{t}$$

17 $${\mathrm{V}}_{\mathrm{t}}^{\text{wt }}\ge {\mathrm{V}}^{\text{cut-out }},\forall \mathrm{t},0$$

18 $${\mathrm{V}}_{\mathrm{t}}^{\mathrm{wt}}\le {\mathrm{V}}^{\text{cut-in }},\forall \mathrm{t},0$$

Wind turbines cannot be operated without risk when the wind speed exceeds the cut-out speed, which is the highest wind speed at which they produce their most electricity. Cut-in speed is the lowest wind speed at which a wind turbine produces the least amount of energy; when wind speeds are below cut-in speed, no energy is produced. As a result, when the wind speed exceeds the speed of cut-out, the wind turbine switches to the OFF status for protection concerns, and in this case, no energy is produced. Generally speaking, the wind speed is higher during the day and lower at night^31^.

#### Solar panel

**Figure 2:**
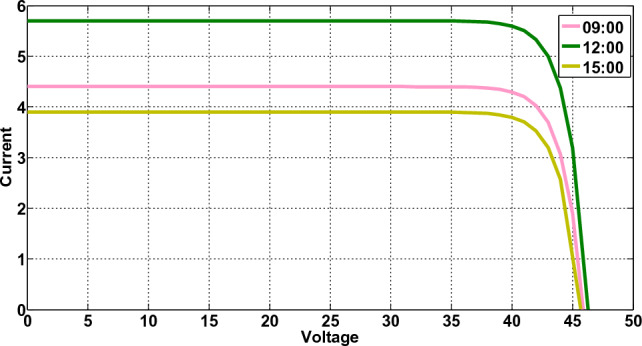
I-V curve.

The suggested DMS makes use of electricity produced by the microgrid during times when it is most expensive to reduce costs while attempting to enhance consumer comfort. Photovoltaic cells convert solar energy into electricity by converting direct current to alternating current via a converter. Current–voltage (I-V) curve and a maximum power point (MPP) that might aid in further optimizing solar cells is produced by integrating performance models for photovoltaic cells. The I-V curve is shown in Fig. 2 and the following equation can be used to estimate how well photovoltaic cells perform:

19 $${i}_{L}-{i}_{S}\mathrm{exp}\left[\alpha \left({v}_{pv}+{R}_{S}i-pv\right)\right]-1{v}_{pv}+{R}_{S}{i}_{pv}/{R}_{Sh}-{i}_{pv}=0, p={V}_{PV}{I}_{PV}$$

where ${i}_{L}$ stands for light current, ${i}_{S}$ is for diode saturation current, $p$ denotes solar panel power generation, ${R}_{Sh}$ stands for stands for and ${R}_{S}$ stands for series resistance. The ideality factor is defined as $\alpha =q/{n}_{s}kT$, where $k=1.38\times {10}^{23}j/K$,$k=1.38\times {10}^{23}j/K$,${n}_{s}$ is the number of solar cells, $T=298\mathrm{ K}$ temperature, and $C$ is the electronic charge. In this study, the generation of power from five $230 W$ solar panels is taken into consideration^29^.

#### Energy storage system

The main objective of the installation of ESS is to maximize the effectiveness of the suggested HEMS. If the cost is minimal, ESS preserves electricity from the commercial grid. It also preserves electricity from the microgrid during peak electricity generation times. In this study, energy storage system is regarded as a shiftable load and discharging and charging can be planned at any time interval in an adaptable manner. Due to safety concerns, ESS can only store a maximum of 90% of the electricity that it can generate (5 kW) in this study^29^.

The maximum and lowest levels of energy storage that ESS enables for the home are 90% and 10%. Only when the microgrid is unable to fulfill demand or electricity tariffs are high does the ESS discharge. The extra electricity is purchased back to the industrial grid when the ESS is at its full storage capacity^32^. Equation (20) represents the stored energy at a specific time slot, considering all the constraints outlined in Eqs. (21) to (23), except for the restrictions on electricity charging and discharging limits.

20 $$\mathrm{SE}(\mathrm{t})=\mathrm{SE}(\mathrm{t}-1)+\mathrm{k}\cdot {\upeta }^{\mathrm{ESS}}\cdot {\mathrm{ES}}^{\mathrm{ch}}(\mathrm{t})-\mathrm{k}\cdot {\mathrm{ES}}^{\mathrm{dis}}(\mathrm{t})/{\upeta }^{\mathrm{ESS}}$$

21 $${\mathrm{ES}}_{\mathrm{t}}^{\mathrm{ch}}<=\mathrm{ES}(\text{ max })$$

22 $${\mathrm{ESS}}_{\mathrm{t}}^{\mathrm{ch}}<\mathrm{ESS}(\text{ upl })$$

23 $${\mathrm{ES}}_{\mathrm{t}}^{\mathrm{dis}}>=\mathrm{ES}(\text{ min })$$

where ${\upeta }^{\mathrm{ESS}}$ is the efficiency of $\mathrm{EES}$, at time $(\mathrm{t})$, the charging of ESS is ${\mathrm{ES}}^{\mathrm{ch}}$, at time $(\mathrm{t})$, the discharging of ESS is ${\mathrm{ES}}^{\mathrm{dis}}$ and $\mathrm{SE}$ shows the stored electricity.

#### Bill calculation and price tariff

**Figure 3:**
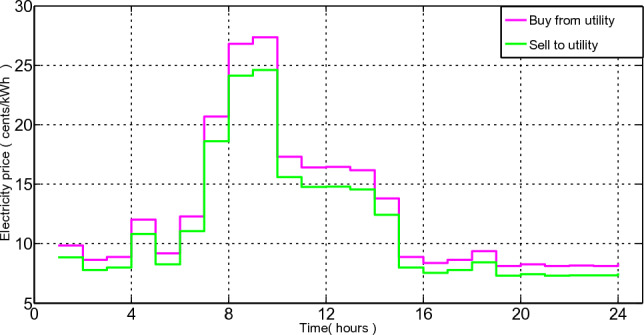
Hourly rates for selling and buying electricity.

For each hour, there are two separate electricity rates: one for buying electricity ${BC}^{buy}$, and the other for selling extra electricity ${BC}^{sell}$ to the commercial grid. However, according to Eq. (24), the hourly selling rate of power is 90% of the hourly purchase rate^33^. Figure 3 shows the electricity selling and buying tariff.

24 $${BC}^{sell}=0.90*{BC}^{buy}$$

A further dynamic feature of our suggested system concept is an electricity pricing. The utility firm offers a variety of electricity rates to encourage customers to control their load requirements. In this study, we calculate the cost of electricity use by taking into account RTP signals.

Consumers reimburse utilities for all costs associated with the electricity they use. Equations (25) and (26) calculate the cost of electricity for an individual time period (hour) with and without mini-grid integration, respectively. The consumer first uses electricity obtained from the mini-grid at any time. The additional electricity needed is then bought from the industrial grid:

25 $${\varsigma }^{t}=((\sum_{dn}{\lambda }_{dn}\times \alpha (t)))-E(t))*{BC}^{buy}(t)$$

26 $${\sigma }^{t}=\sum_{dn}{\lambda }_{dn}\times \rho \left(t\right)\times \alpha \left(t\right)$$

Similarly, Eqs. (27) and (28), which calculate the overall price per day with and without a microgrid, respectively:

27 $${\varsigma }^{total}= \sum_{t=1}^{24}((\sum_{dn}{\lambda }_{dn}\times \alpha (t))-E(t))*{BC}^{buy}(t)$$

28 $${\delta }^{total}=\sum_{t=1}^{24}(\sum_{dn}{\lambda }_{dn}\times \rho \left(t\right)\times \alpha \left(t\right))$$

At the beginning of each hour, the choice is made not to store, buy, or sell the electricity. The smart house attempts to buy electricity for load needs when the price is lower. Electricity produced by mini-grids is retained in ESS for potential later exchanging. The smart house uses ESS or the mini-grid to fulfill its load during ON-peak times, and any extra power is traded back to the industrial grid according to Eq. (29):

29 $${\eta }^{sell}\left(t\right)=(\sum_{dn}{\lambda }_{Dn}\times \alpha (t))-[E\left(t\right)+ESS]$$

30 $${\eta }^{sell}\left(t\right)=\left\{\begin{array}{ll}{\eta }^{sell}\left(t\right),& \text{ If }{\eta }^{sell}\left(t\right) <0,\\ 0,& \text{ otherwise}\end{array}\right.$$

Equation (31) calculates the total quantity of electricity exported to the commercial grid:

31 $${\eta }^{t}=\sum_{t=1}^{T}[{\eta }^{sell}\left(t\right)]$$

Equations (32) and (33), which show the day total and hourly profits from a commercial grid, respectively:

32 $${\k{e} }^{earn}\left(t\right)={\eta }^{sell}\left(t\right)*{BC}^{sell}(t)$$

33 $${\k{e} }^{t}=\sum_{t=1}^{T}[{\eta }^{sell}\left(t\right)*{BC}^{sell}(t)]$$

#### Constraints

Constraints are an essential component of optimization problems, as they define the limitations and boundaries within which a solution must operate. They play a critical role in guiding the optimization process and ensuring that the solutions generated are not only optimal but also feasible. In smart home, constraints serve to reflect real-world limitations, making sure that the solutions found are not only mathematically optimal but also practical and applicable to the problem at hand. Balancing the optimization objectives with these constraints is a crucial aspect of the optimization process.

Constraints in this study encompass various factors and limitations. They include device-specific power requirements, scheduling restrictions. In addition to these constraints, wind turbines have specific limitations that must be considered. For instance, they cannot operate safely when wind speeds exceed the cut-out speed, which is the highest wind speed at which they can efficiently generate electricity. Conversely, the cut-in speed is the lowest wind speed at which a wind turbine can produce energy, with no energy generation occurring when wind speeds are below this threshold. Equations (16)–(18) present all restrictions for wind turbines.

Table 2 displays several metrics for our studies, where ${\mathrm{ESS}}^{\mathrm{CAP}}$, ${\mathrm{solar}}^{\mathrm{cap}}$, and ${\mathrm{wind}}^{\mathrm{cap}}$ represent the average hourly energy production from ESS, solar panel, and wind turbine respectively. ${\mathrm{V}}^{\mathrm{cut}-\mathrm{off}}$ and ${\mathrm{V}}^{\mathrm{cut}-\mathrm{in}}$ depict the wind speed at the cut-out and cut-in points when the wind turbine produces the most and least energy, respectively. Also, managing the stored energy in a certain period of time requires restrictions to ensure safe and optimal operation, and these restrictions are represented mathematically using Eq. (21) to Eq. (23).

**Table 2:** Parameters for case studies.

Parameters	${\mathrm{ESS}}^{\mathrm{CAP}}$	${\mathrm{solar}}^{\mathrm{cap}}$	${\mathrm{wind}}^{\mathrm{cap}}$	${\mathrm{V}}^{\mathrm{cut}-\mathrm{off}}$	${\mathrm{V}}^{\mathrm{cut}-\mathrm{in}}$	${\eta }^{\mathrm{ESS}}$	SOC
Values	5 KW	1 KW	2 KW	25	5	95%	90%

## Proposed algorithm

Every house in the smart grid environment has a smart meter installed, and the smart meter is further linked to the energy management controller. Only smart meters allow for 2-way communication among electricity users and utilities. In this work, we examine heuristic methods for solving trading and scheduling issues. The use of the manta ray foraging optimization (MRFO) algorithm and Long term memory MRFO (LMMRFO) algorithm aims to maximize profits while minimizing costs and PAR. In benchmark tests, MRFO has exhibited competitive performance compared to other optimization algorithms, establishing itself as a reliable choice for solving complex optimization problems. While the effectiveness of MRFO algorithm in search mechanisms is evident, there remain certain areas where enhancements can boost its efficiency in tackling challenging optimization problems, specifically the scheduling and trading problem. This is imperative as the current algorithm may overlook critical search regions^41^. Additionally, the MRFO algorithm exhibits shortcomings, such as insufficient exploitation capability, reduced population diversity, and a susceptibility to getting stuck in local optima. These limitations primarily stem from an imbalance in the algorithm's exploitation and exploration of the search space^42^. To elevate algorithm performance and rectify the equilibrium between exploitation and exploration capabilities, this paper introduces LMMRFO, which incorporates a long-term memory strategy. This strategy is designed to augment the algorithm's exploitation ability, addressing the issue of slow convergence. These characteristics make LMMRFO technique a promising technique for efficiently scheduling devices for power trading, facilitating optimal energy management in smart homes.

This section first provides a thorough introduction of MRFO and LMMRFO, followed by an explanation of how MRFO and LMMRFO work in relation to the benchmark test functions and our scheduling and trading problem.

### Manta Ray foraging optimization

The MRFO was introduced in 2020 by Zhao et al.^34^. The MRFO algorithm is depending on simulating the behaviors of the clever actions of manta rays. Chain foraging, cyclone foraging, and somersault foraging, three distinct foraging techniques used by manta rays, are imitated in this work to create an effective optimization paradigm for resolving various optimization issues.

**Figure 4:**
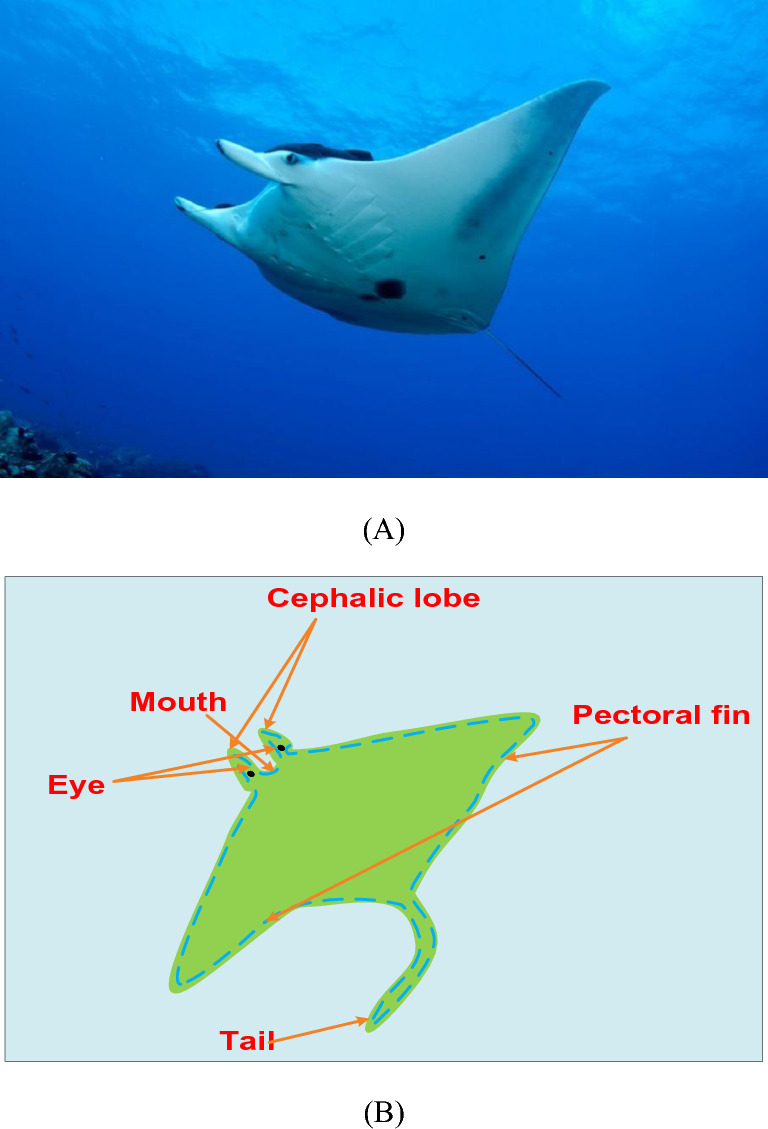
(**A**) A manta ray foraging, and (**B**) a manta ray's structure.

Manta rays look awful, but they are sophisticated creatures. They rank among the biggest marine animals ever discovered. Manta rays gracefully swim while using their pectoral fins, which have a flat body from top to bottom. A pair of cephalic lobes that extend in front of their enormous terminal mouths are also present. Figure 4A features an image of a manta ray engaged in foraging, captured by Swanson Chan on Unsplashed, while Fig. 4B depicts the anatomical structure of a manta ray. Manta rays feed on plankton, which consists mainly of tiny aquatic organisms, despite lacking sharp teeth. During foraging, they employ their horn-shaped cephalic lobes to draw both water and prey into their mouths.

#### Mathematical model

Chain harvesting, cyclone foraging, and somersault foraging are the three foraging techniques that served as the inspiration for MRFO. Below is a description of the mathematical models.

##### Chain harvesting

**Figure 5:**
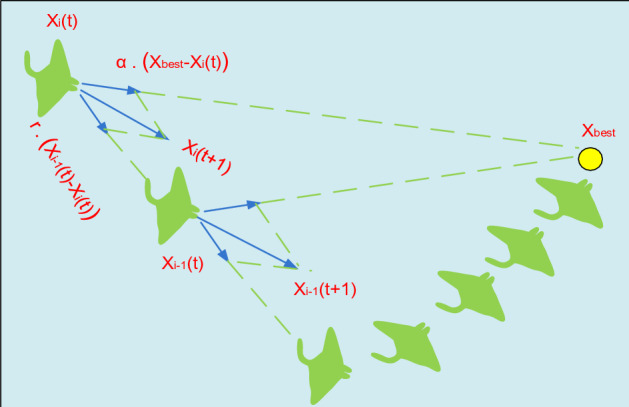
Chain Harvesting.

Manta rays can see where plankton is in MRFO and swim toward it. A place is better the higher the concentration of plankton there is. The optimum answer may not be known, but according to MRFO, the plankton with a high concentration that manta rays desire to approach and feed is the best one so far discovered. Manta rays align themselves in a foraging chain from head to tail. Everyone but the first person moves toward the meal and the person in front of it. In other words, each person gets updated throughout each iteration with the best answer so far and the solution in front of it. The following is a representation of this chain foraging mathematical model:

34 $$x_{i}^{d} \left( {t + 1} \right) = \left\{ {\begin{array}{*{20}c} {x_{i}^{d} \left( t \right) + r \cdot \left( {x_{best}^{d} \left( t \right) - x_{i}^{d} \left( t \right)} \right) + \alpha \cdot \left( {x_{best}^{d} \left( t \right) - x_{i}^{d} \left( t \right)} \right)} & {\text{ i = 1,}} \\ {x_{i}^{d} \left( t \right) + r \cdot \left( {x_{i - 1}^{d} \left( t \right) - x_{i}^{d} \left( t \right)} \right) + \alpha \cdot \left( {x_{best}^{d} \left( t \right) - x_{i}^{d} \left( t \right)} \right)} & {{{i = 2,}} \ldots {\text{N}}} \\ \end{array} } \right.$$

35 $$\alpha =2.r\sqrt{\left|\mathrm{log}(r)\right|}$$

where,$d$ is dimensions, $t$ is time, the individual $i$ position is ${x}_{i}^{d}$, $r$ is a random vector with range between 1 and 0, $\alpha$ is a weight coefficient, and ${x}_{best}^{d}$ is the concentrated plankton. This foraging activity is shown in Fig. 5.

##### Cyclone foraging

**Figure 6:**
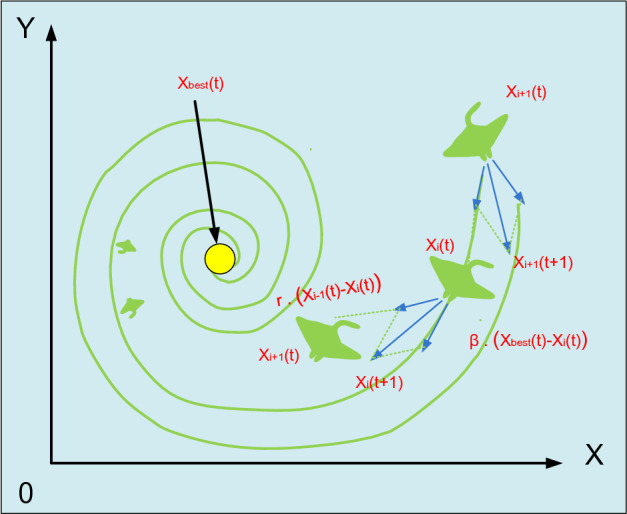
Foraging activity of cyclones.

A school of manta rays will create a long harvesting chain and travel in a spiral toward the food when they spot a patch of plankton in deep water. WOA employs a similar spiral foraging technique^35^. However, in manta ray swarms, the cyclone foraging method, each manta ray swims toward the one in front of it in addition to spiraling towards the food. In other words, manta rays perform foraging in swarms that create a spiral. The foraging behavior of a cyclone is depicted in Fig. 6. A creature only moves in a spiraling motion in the direction of the food as opposed to just following the one in front of it. The mathematical formula that describes how manta rays travel in a spiral pattern in two dimensions, it is calculated by:

36 $$\left\{\begin{array}{c}{X}_{i}\left(t+1\right)={X}_{best}+r.\left({X}_{i-1}\left(t\right)-{X}_{i}\left(t\right)\right)+{e}^{bw}.\mathrm{cos}\left(2\pi w\right).({X}_{best}-{X}_{i}(t))\\ {Y}_{i}\left(t+1\right)={Y}_{best}+r.\left({Y}_{i-1}\left(t\right)-{Y}_{i}\left(t\right)\right)+{e}^{bw}.\mathit{sin}\left(2\pi w\right).({Y}_{best}-{Y}_{i}\left(t\right))\end{array}\right.$$

37 $$x_{i}^{d} \left( {t + 1} \right) = \left\{ {\begin{array}{*{20}c} {x_{best}^{d} \left( t \right) + r \cdot \left( {x_{best}^{d} \left( t \right) - x_{i}^{d} \left( t \right)} \right) + \beta \cdot \left( {x_{best}^{d} \left( t \right) - x_{i}^{d} \left( t \right)} \right),} & {\text{ i = 1}} \\ {x_{best}^{d} \left( t \right) + r \cdot \left( {x_{i - 1}^{d} \left( t \right) - x_{i}^{d} \left( t \right)} \right) + \beta \cdot \left( {x_{best}^{d} \left( t \right) - x_{i}^{d} \left( t \right)} \right),} & {{{i = 2, \ldots N}}} \\ \end{array} } \right.$$

where *w* and $r$ are random numbers within the range between 1 and 0. $\beta$ is the weight coefficient.

All individuals conduct their searches at random, using the food as their point of reference. As a result, the area with the optimal solution thus far is well-utilized by cyclone foraging. The exploration is also much enhanced by this behavior. By designating a new random position throughout the whole search area as their reference position, we may drive each person to look for a new position far from the current best one. The mathematical equation for this mechanism, which primarily focuses on exploration and allows MRFO to conduct a thorough global search, it is calculated by:

38 $${x}_{rand}^{d}={Lb}^{d}+r.({Ub}^{d}-{Lb}^{d})$$

39 $$x_{i}^{d} \left( {t + 1} \right) = \left\{ {\begin{array}{*{20}c} {x_{{rand}}^{d} \left( t \right) + r \cdot \left( {x_{{rand}}^{d} \left( t \right) - x_{i}^{d} \left( t \right)} \right) + \beta \cdot \left( {x_{{rand}}^{d} \left( t \right) - x_{i}^{d} \left( t \right)} \right)} & {{\text{~i = 1}}} \\ {x_{{rand}}^{d} \left( t \right) + r \cdot \left( {x_{{i - 1}}^{d} \left( t \right) - x_{i}^{d} \left( t \right)} \right) + \beta \cdot \left( {x_{{rand}}^{d} \left( t \right) - x_{i}^{d} \left( t \right)} \right)} & {{{i = 2, \ldots \ldots N}}} \\ \end{array} } \right.$$

where ${x}_{rand}^{d}$ is a random position generated at random in the search space, and the upper and bottom bounds of the dimension are ${Lb}^{d}$ and ${Ub}^{d}$.

##### Foraging in somersault

The location of the food is seen as a pivot in this behavior. Each person usually swims back and forth around the pivot before somersaulting into a different position. As a result, they constantly update their positions in relation to the best position thus far. The following can be done to generate the mathematical model:

40 $${x}_{i}^{d}\left(t+1\right)={x}_{i}^{d}\left(t\right)+S.\left({r}_{2}.{x}_{best}^{d}-{r}_{3}.{x}_{i}^{d}\left(t\right)\right), i=1,\dots \dots .N$$

where $S$ is the somersault factor that determines the manta rays' somersault range and ${r}_{2}$ and ${r}_{3}$ are two random values between 0 and 1.

**Figure 7:**
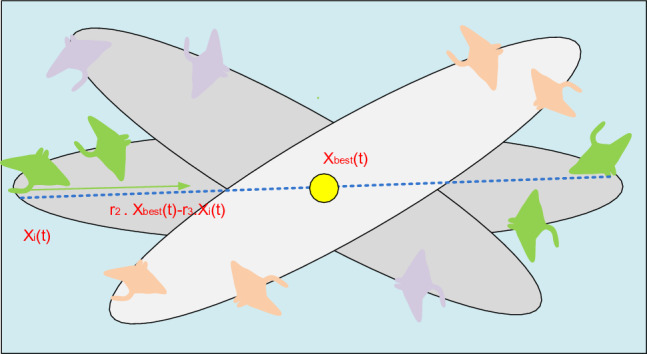
MRFO's somersault foraging technique.

As can be seen from Eq. (40), each individual has the ability to relocate to any position inside a new search area that is situated among its symmetrical position around the best position discovered and its current position thus far. The disturbance on the present position decreases as the separation among the best position and the individual position thus far decreases. Each person eventually gets closer to the ideal outcome in the search area. when a result, when iterations rise, the somersault foraging range adaptably decreases. The somersault foraging behavior of MRFO is depicted in Fig. 7.

**Figure 8:**
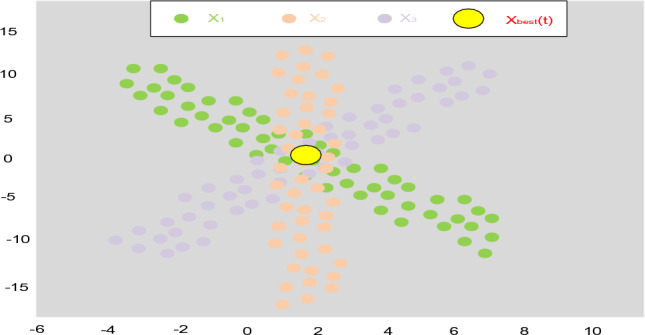
Three individuals’ somersault foraging behavior.

According to Eq. (40), Fig. 8 demonstrates that three individuals developed 100 times in the search space. As the distance shrinks, the sampled points become sparser and randomly spread between their symmetrical places around the current coordinates. The nearby dense sites can significantly aid in exploitation, while the nearby sparse points can greatly aid in exploration.

### Long term memory Manta Ray foraging optimization

Though the MRFO optimization method shows promise, there is room for further improvement to achieve best results. Like numerous other metaheuristic algorithms, MRFO technique relies on searching around a single global best position. However, this approach of having a single reference point for the entire population possibly not ensure concourse to the global optimal point. The reliance on a single global best position often leads to premature convergence in metaheuristic techniques. To address this limitation, this research introduces the concept of long-term memory in MRFO. By incorporating long-term memory, the population of individuals can make decisions based on multiple past experiences. This approach offers a broader perspective of many potentially successful points, thereby mitigating risk of premature stagnation. The proposed method, called Long-Term Memory MRFO (LMMRFO), introduces an additional parameter known as Memory Length. Memory Length (ML) is a user-defined control factor that determines how many past experiences population or swarm can recall at any given time^36^. By incorporating long-term memory into MRFO, LMMRFO enhances the algorithm's ability to explore diverse solutions and avoid premature convergence.

The updating process of the long-term memory in LMMRFO follows a First-In-First-Out (FIFO) queue mechanism. The memory stores the ML best locations discovered thus far. In the FIFO approach, when a new item is added, the oldest item is removed to maintain the queue's length. In the case of MRFO, during each iteration t, the memory is modified by adding the most recent optimal point found while deleting oldest entry. Once the memory is modified, swarm determines its subsequent step by selecting single item from the long-term memory. The choice is made probabilistically, using a probability calculation for each item in memory, denoted as pi for the ith item, as shown in Eq. (41):

41 $$\mathrm{pi}=\frac{\mathrm{f}({\mathrm{x}}_{\mathrm{i}}^{\mathrm{d}})}{\sum_{\mathrm{j}=1}^{\mathrm{ML}}\mathrm{f}({\mathrm{x}}_{\mathrm{j}}^{\mathrm{d}})}$$

In Eq. (41), $\mathrm{f}({\mathrm{x}}_{\mathrm{j}}^{\mathrm{d}})$ or $\mathrm{f}({\mathrm{x}}_{\mathrm{i}}^{\mathrm{d}})$ represents the fitness value of the jth or ith clause in the long-term memory. After computing the probability of selection for each item within the long-term memory, the Roulette Wheel Selection method is employed to execute the selection process. Upon selecting a clause from the long-term memory, this method can be applied in all equations for updating positions. In LMMRFO, in place of using the position of the single global optimal, ${\mathrm{x}}_{\mathrm{best}}$, as in MRFO, LMMRFO employs ${\mathrm{x}}_{\mathrm{best}}^{\mathrm{k}}$, which corresponds to the kth global optimal position stored in the long-term memory. K is chosen through the Roulette Wheel Selection method. As a result, each position modifies equations in LMMRFO remain the same as MRFO, with the exception of replacing ${\mathrm{x}}_{\mathrm{best}}$ with ${\mathrm{x}}_{\mathrm{best}}^{\mathrm{k}}$.

**Figure 9:**
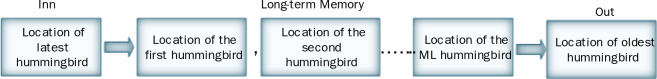
Long-term memory update process.

**Figure 10:**
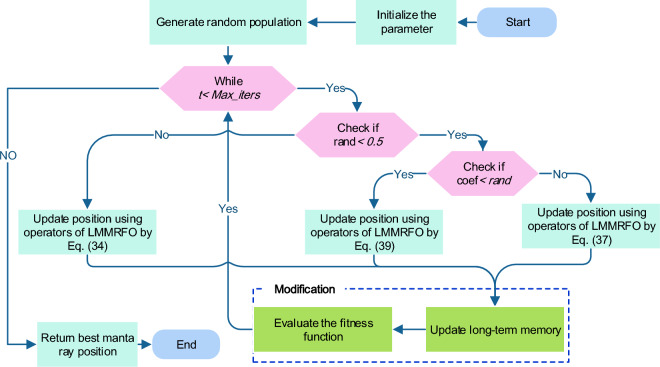
Flow chart of LMMRFO algorithm.

The process of modifying the long-term memory is illustrated in Fig. 9. As mentioned previously in this section, LMMRFO gives a updating to the original algorithm. The emphasizes the importance of conducting thorough analyses of metaheuristic algorithms to achieve efficient results, rather than introducing new algorithms frequently when numerous alternatives already exist. The flow chart of LMMRFO algorithm is illustrated in Fig. 10.

## Benchmark functions

In this section, we demonstrate the effectiveness of the LMMRFO technique by evaluating its performance on seven benchmark functions. The mathematical expressions for these test functions can be found in Table^37^. These benchmark experiments were conducted using MATLAB (R2016a) on a computer equipped with an Intel(R) Core i5-4210U CPU running at 2.40 GHz and 8 GB of RAM. This study utilizes seven widely recognized benchmark test functions to assess and compare the performance of the LMMRFO technique. All the metaheuristic methods discussed in this paper are subjected to a uniform maximum iteration limit of 200 iterations, along with a consistent population size of 50. In this section, we compare the LMMRFO technique with four recently proposed techniques, namely GTO^38^, HPO^39^, BWO^40^, and MRFO algorithms.

**Table 3:** Benchmark functions.

Name	Function	Dim	Range	f_min_
Sphere	${F}_{1}\left(x\right)={\sum }_{i=1}^{N}{x}_{i}^{2}$	30	[− 100, 100]	0
Schwefel 2.22	${F}_{2}\left(x\right)={\sum }_{i=1}^{N}\left|{x}_{i}\right|+{\prod }_{i=1}^{N}\left|{x}_{i}\right|$	30	[− 10, 10]	0
Schwefel 1.2	${F}_{3}\left(x\right)={\sum }_{i=1}^{N}{\left({\sum }_{j-1}^{i}{x}_{j}\right)}^{2}$	30	[− 100, 100]	0
Schwefel 2.21	${F}_{4}\left(x\right)={max}_{i}\left\{\left|{x}_{i}\right|,1\le i\le N\right\}$	30	[− 100, 100]	0
Rosenbrock	${F}_{5}\left(x\right)={\sum }_{i=1}^{N-1}[100{\left({x}_{i+1}-{x}_{i}^{2}\right)}^{2}+{\left({x}_{i}-1\right)}^{2}$	30	[− 30, 30]	0
Step	${F}_{6}\left(x\right)={\sum }_{i=1}^{N}{\left(\left|{x}_{i}+0.5\right|\right)}^{2}$	30	[− 100, 100]	0
Quartic	${F}_{7}\left(x\right)={\sum }_{i=1}^{N}{ix}_{i}^{4}+random[\mathrm{0,1}]$	30	[− 1.28, 1.28]	0

This study establishes the supremacy of the obtained solution using the mean value and standard deviation. An algorithm with lower mean value and standard deviation demonstrates robust global optimization capabilities and greater stability. The statistical results derived from the LMMRFO algorithm and four well-known algorithms, applied to solve seven benchmark functions, are presented in Table 4. As depicted in Table 4, the LMMRFO technique outperforms other evaluated methods across the majority of benchmark functions in terms of the mean value. The data clearly indicates that the LMMRFO algorithm consistently achieves more favorable solutions compared to recently proposed techniques for solving various benchmark functions. Additionally, it is evident that the LMMRFO approach surpasses GTO, HPO, BWO, and MRFO techniques in addressing benchmark functions. This analysis underscores the efficiency of the LMMRFO algorithm.

**Table 4:** the statistical results of seven benchmark functions by the LMMRFO algorithm and other well-known techniques.

Function		LMMRFO	MRFO	GTO	HPO	BWO
F1	Best	2.1E−202	8.8E−180	1.3E−176	1.17E−77	1.5E−111
	Average	3.3E−190	3E−168	9.1E−152	1.49E−69	3.8E−106
	Median	4.5E−196	8.2E−174	4.2E−170	3.09E−72	6.8E−108
	Worst	3.1E−189	5.3E−167	1.8E−150	1.92E−68	2.5E−105
	std	0	0	4.1E−151	4.51E−69	6.8E−106
	Rank	1	2	3	5	4
F2	Best	3.8E−100	5.73E−92	2.96E−85	5.41E−41	1.95E−57
	Average	4.94E−98	2.78E−85	1.44E−80	2.69E−38	2.56E−54
	Median	1.63E−98	8.8E−87	5.06E−82	1.64E−39	1.15E−54
	Worst	2.98E−97	3.19E−84	9.29E−80	2.64E−37	1.18E−53
	std	9.12E−98	7.82E−85	2.93E−80	6.13E−38	3.58E−54
	Rank	1	2	3	5	4
F3	Best	1.4E−184	1.7E−172	3.8E−167	4.64E−68	6.4E−104
	Average	1.2E−170	5.2E−162	3.9E−149	1.72E−59	5.5E−100
	Median	2E−176	4.9E−167	9.3E−157	5.21E−63	1.2E−101
	Worst	8.8E−170	8.3E−161	7.4E−148	3.12E−58	7.3E−99
	std	0	1.8E−161	1.7E−148	6.95E−59	1.6E−99
	Rank	1	2	3	5	4
F4	Best	1.4E−100	3.32E−88	8.11E−88	2.1E−35	9.97E−55
	Average	9.68E−95	1.1E−83	1.3E−80	7.25E−32	2.03E−52
	Median	1.36E−96	1.57E−84	8.8E−83	1.31E−32	5.03E−53
	Worst	9.48E−94	1.44E−82	1.37E−79	7.27E−31	1.38E−51
	std	2.99E−94	3.22E−83	3.51E−80	1.69E−31	4.03E−52
	Rank	1	2	3	5	4
F5	Best	2.01E−05	24.05046	4.82E−05	23.80007	0.000406
	Average	0.000206	24.74472	3.890629	24.34554	0.010977
	Median	0.000109	24.73866	0.003429	24.16784	0.006422
	Worst	0.000617	25.81043	26.5186	25.98622	0.05599
	std	0.000221	0.428115	9.448749	0.511546	0.015734
	Rank	1	5	3	4	2
F6	Best	1.74E−08	4.8E−06	1.28E−07	3.7E−07	2.47E−08
	Average	1.79E−07	2.36E−05	0.000152	0.000811	5.36E−07
	Median	1.4E−07	1.09E−05	2.01E−05	1.8E−06	3.77E−07
	Worst	5.4E−07	0.000139	0.001805	0.015867	1.4E−06
	std	1.71E−07	3.13E−05	0.0004	0.003544	4.28E−07
	Rank	1	3	4	5	2
F7	Best	2.28E−05	1.75E−05	5.83E−06	2.71E−05	2.65E−06
	Average	0.000166	0.000226	0.000168	0.000529	0.000257
	Median	0.000146	0.00019	0.000152	0.000167	0.000225
	Worst	0.000398	0.000832	0.000421	0.001566	0.000674
	std	0.000136	0.000189	0.00012	0.000569	0.000205
	Rank	1	3	2	5	4
Average Rank		1	2.714286	3	4.857143	3.428571
Final ranking		1	2	3	5	4

The tied rank method is a statistical approach used to compare the performance of multiple techniques when there are ties in the performance metric, signifying those two or more observations share the same value. After assigning ranks, the rank sums for each algorithm are compared. The algorithm with the lowest rank sum is deemed to have performed better than the others. The data presented in Table 4 unequivocally demonstrates that the LMMRFO technique exhibits superior performance across the majority of the 7 benchmark optimization problems, as evidenced by its ranking order. In second and third positions are the MRFO and GTO algorithms, both displaying robust efficacy. This collective evidence establishes the LMMRFO technique as a highly effective algorithm for successfully identifying optimal solutions within this category of problems.

**Figure 11:**
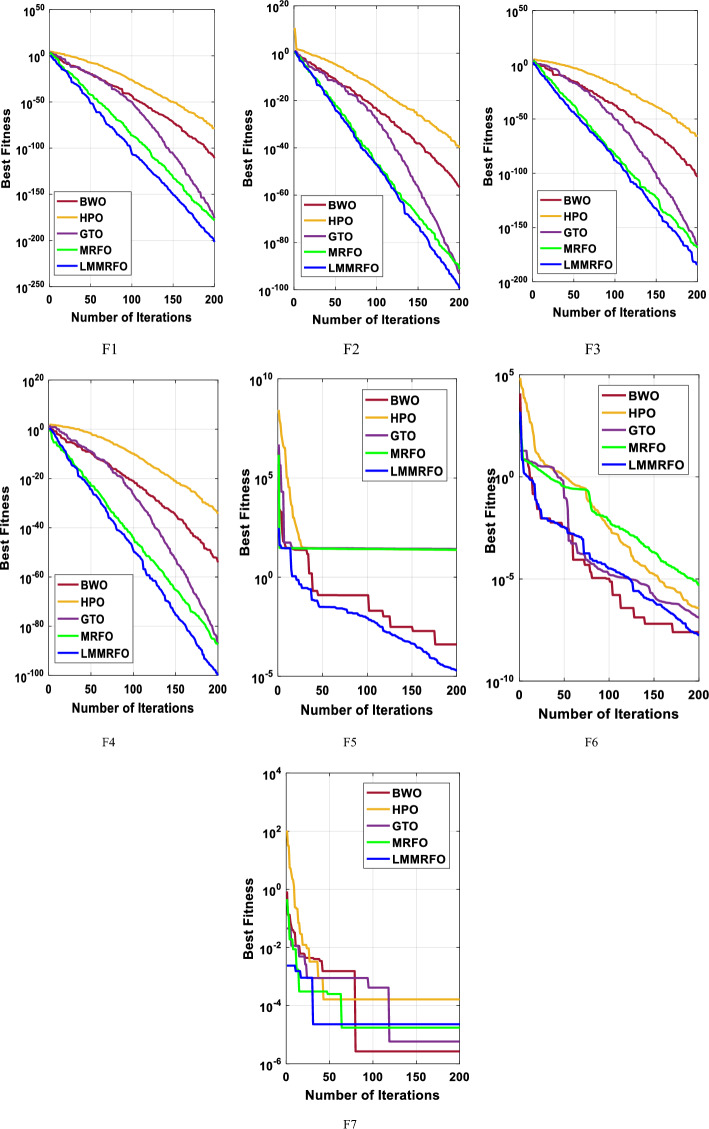
The convergence characteristics of the studied techniques for the benchmark functions.

Moreover, the convergence curves unmistakably highlight the consistent superiority of the LMMRFO technique when compared to other methods across the majority of benchmark functions. This underscores its robustness and adaptability in addressing a diverse spectrum of optimization problems. The exceptional performance of the LMMRFO technique can be attributed to its effective amalgamation of the MRFO algorithm and Long-Term Memory strategy. This combination empowers it to adeptly navigate the search space, exploit promising regions, and achieve faster convergence with superior solutions. Furthermore, the convergence curves, depicted in Fig. 11, not only confirm the rapid convergence of the LMMRFO technique but also its ability to sustain stable and reliable performance throughout the optimization process. This is a pivotal trait for an optimization algorithm, assuring that it consistently identifies optimal solutions without being trapped in local minima. Additionally, the experimental findings indicate that the LMMRFO technique exhibits resilience to variations in optimization parameters, such as population size and crossover probability. This resilience implies that the LMMRFO algorithm can be seamlessly applied to diverse problem domains without the need for extensive parameter fine-tuning.

**Figure 12:**
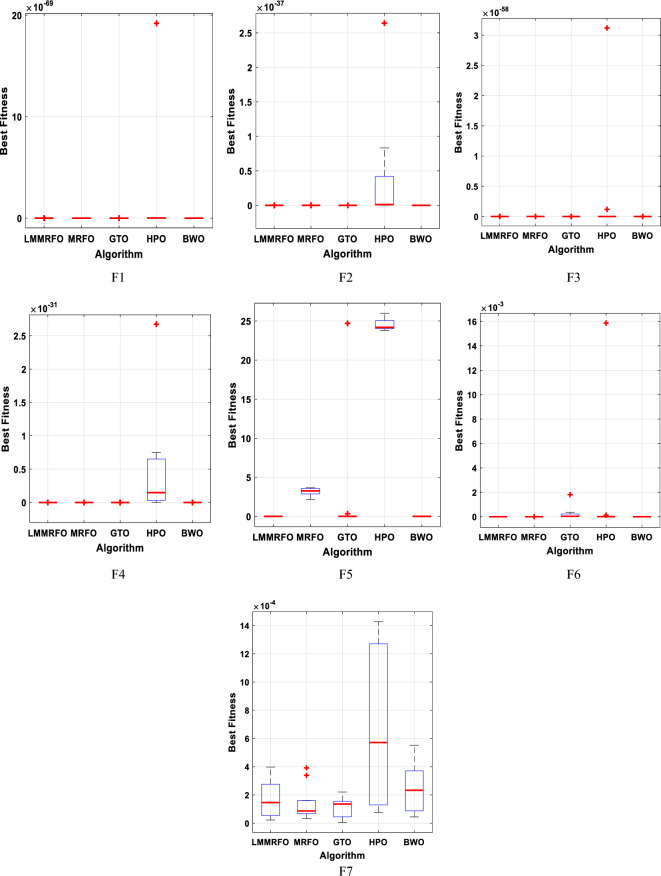
Boxplots of the studied techniques for the benchmark functions.

In summary, these results establish the LMMRFO technique as a potent and versatile optimization method capable of efficiently achieving optimal solutions for a wide range of real-world optimization challenges. Its rapid convergence, consistent performance, and parameter robustness make the LMMRFO algorithm an appealing choice for both practitioners and researchers. The numerical data is visually represented in box plots, illustrating the diverse optimal values obtained across multiple runs for each specific algorithm. Figure 12 displays these box plots for seven benchmark functions, utilizing data collected from 30 individual iterations. Box plots excel at depicting data distribution, offering a clear means to emphasize data consistency. Upon examination of Fig. 12, it becomes evident that the box plots for the LMMRFO technique showcase narrower spreads and rank among the lowest values across most functions. These visual representations serve as powerful tools for assessing the performance of the nonlinear system, providing a clear contrast between different techniques. The results unequivocally highlight the superior performance of the LMMRFO method.

### Wilcoxon's rank test results

In this subsection, we conduct a further statistical analysis to assess the variances between LMMRFO and other techniques using the Wilcoxon rank-sum test (WRST), a paired assessment method employed to identify significant differences between the two techniques. The results of these tests, conducted at a significance level of α = 0.05, are presented in Table 5. In the table, symbols like "+/=/−" indicate whether LMMRFO performs better, similarly, or worse than the compared technique. Furthermore, the table provides statistical insights into LMMRFO's performance across different dimensions and functions, revealing whether it outperforms, performs similarly, or lags behind the comparison algorithm.

**Table 5:** Statistical results of the Wilcoxon rank-sum test.

LMMRFO vs	MRFO		GTO		HPO		BWO	
Function	P	Winner	P	Winner	P	Winner	P	Winner
F1	1.83E−04	+	1.83E−04	+	1.83E−04	+	1.83E−04	+
F2	1.83E−04	+	1.83E−04	+	1.83E−04	+	1.83E−04	+
F3	1.13E−02	+	1.83E−04	+	1.83E−04	+	1.83E−04	+
F4	1.83E−04	+	1.83E−04	+	1.83E−04	+	1.83E−04	+
F5	1.83E−04	+	5.80E−03	+	1.83E−04	+	3.30E−04	+
F6	1.83E−04	−	7.69E−04	+	2.46E−04	+	9.11E−03	+
F7	9.70E−01	=	2.73E−01	=	3.12E−02	+	2.12E−01	=
WRST (+/=/−)	5/1/1		6/1/0		7/0/0		6/1/0	

It is worth noting that LMMRFO exhibits superior statistical performance in F1–F7 with Dim = 30 when compared to other techniques, affirming its significant dominance across most functions. Consequently, we can confidently conclude that the proposed LMMRFO technique demonstrates the best overall performance when compared to other methods.

### Friedman’s rank test results

Table 6 presents the statistical results obtained through Friedman tests^43^ conducted on seven benchmark functions employing the analyzed algorithms. In this analysis, a lower ranking value signifies a more superior algorithm performance. The results reveal a distinct ranking order among the five techniques, which is as follows: LMMRFO, MRFO, GTO, HPO, and BWO. This ranking order provides valuable insight into the relative performance of these algorithms across the benchmark functions, with LMMRFO emerging as the top-performing technique, followed by MRFO, GTO, BWO, and HPO in descending order. These findings offer a comprehensive view of how the algorithms fare in comparison to one another across various test functions.

**Table 6:** Friedman test for the five algorithms.

Function	LMMRFO	MRFO	GTO	HPO	BWO
F1	1	2.5	2.5	5	4
F2	1	2	3	5	4
F3	1.2	1.8	3	5	4
F4	1	2	3	5	4
F5	1.1	3.9	2.5	4.9	2.6
F6	2.1	1	4.8	4.1	3
F7	2.8	2.4	2.5	3.9	3.4
Mean ranks	1.457143	2.228571	3.042857	4.7	3.571429

## Scenario studies

Three scenario studies are offered in this paper to show the effectiveness of our suggested system from the viewpoints of the user/smart house as well as the commercial grid. In the initial scenario work, house lacks intelligence; in other words, we take into account a typical house and the electrical system. Without being aware of the electricity rate, the house purchases and uses conventional electricity. In the other two instances, homes with various features such as the integration of a wind turbine, solar panels, and ESS are taken into account and compared to one another from the viewpoints of the customer and the electrical network. The smart house capable buy, storage, sell or move the load during the transient phase of each case study, which is when each smart house takes autonomous decisions in relation to the commercial grid. The proposed system's overall performance will eventually stabilize.

## The results

The results of the simulation are displayed to determine the power exchange and best scheduling for the home. We conduct simulations several times before averaging the findings from 20 runs. To evaluate and validate our suggested method, two algorithms LMMRFO are MRFO used. We imagine a smart home with 12 distinct smart appliances, each of which has a different operational times and power rating as described in Table 2. These appliances are divided into three different groups as well. Because base-load appliances cannot be moved and must be turned ON in accordance with user choices, they may not help reduce power prices or PAR. In this paper, the operation period is divided into 24 1-h slots starting at 6 a.m. and ending at 6 a.m. the following day. For the purpose of calculating electricity costs, we take into account RTP signals, which are shown in Fig. 3. On a computer system with a 2.3 GHz processor, 4 GB RAM, and Windows 7 Operating System, simulations and experiments of our suggested model were run. Additionally, this approach uses MATLAB as a simulator.

### Scenario 1: House without Microgrid and Energy Management

**Figure 13:**
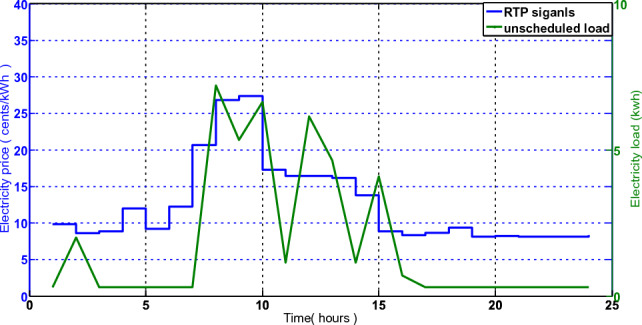
Electricity consumption and Pricing signals.

**Figure 14:**
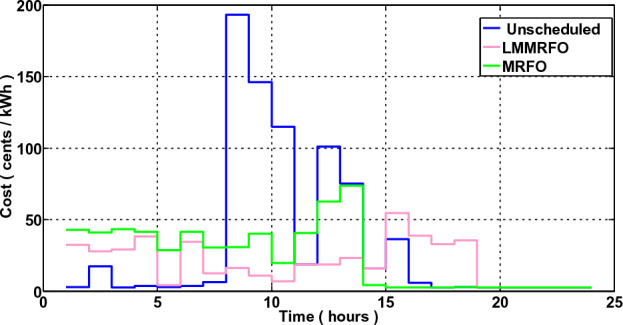
Cost of power per hour.

**Figure 15:**
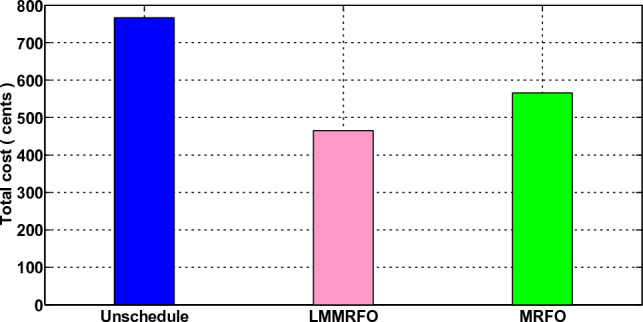
Total cost.

In this scenario, the operational behavior of a standard house in the absence of microgrid connection and ESS was investigated. The traditional house is unable to control its electricity usage, and it has no extra energy that could be sold back to the power system. The house is also powerless to decide how much electricity to use. It purchases and uses electricity without thoughtful planning, disregarding the tariff rates or any other factor. The electricity normal residences purchase from the electric grid is shown in Fig. 13 along with price signals. The findings clearly show that the typical house does not take into consideration pricing tariffs and uses electricity without careful consideration. The typical residence makes its electricity purchases in time slots 10 and 9 when the price is at its highest and also causes peaks because of its highest electricity usage. As a result, the typical residence would incur the highest electricity expenses due to improper use of electricity. Figures 14 and 15, respectively, show the hourly and total electricity costs vs unscheduled usage. However, the findings in this section serve as a starting point for our subsequent comparisons.

### Scenario 2: Home without Microgrid but With Energy Management

**Figure 16:**
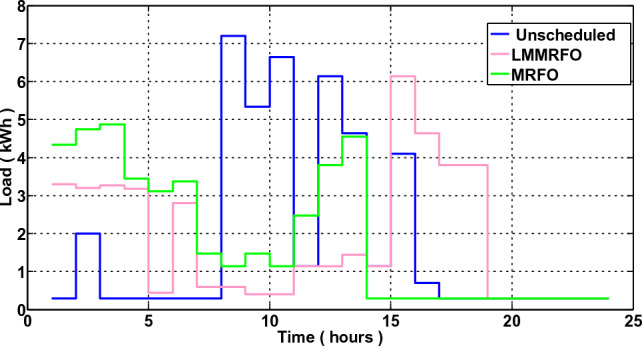
The amount of electricity consumed during each hour of the day.

**Figure 17:**
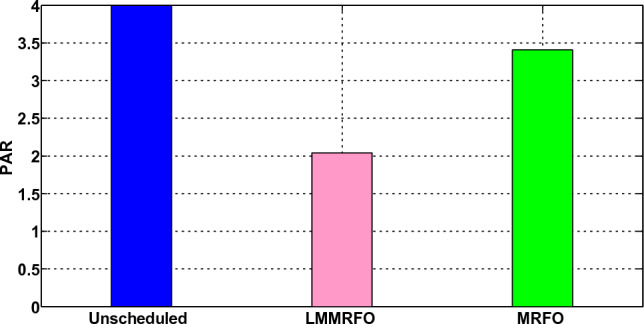
Peak to average ratio.

In this scenario, a home's energy management is taken into account, and a smart home is able to control its electricity usage. In this case, EMC is in place, and EMC adjusts the load in accordance with the load information and price. It's possible that the shiftable devices moved from ON-peak to Off-peak times. The hourly electricity usage with and without energy management using LMMRFO and MRFO is shown in Fig. 16. The outcome shows that during ON-peak times, without energy management, electricity consumption is high, but during ON-peak times with LMMRFO and MRFO, electricity consumption is low. As seen in Fig. 17, our suggested techniques effectively move the load while reducing PAR. The comparison to MRFO and an unplanned case, LMMRFO performs well in reducing PAR. The PAR is 3.41 in the case of using MRFO, but in the case of using LMMRFO it is 2.04. Using MRFO and LMMRFO, the PAR is reduced by 15% and 49%, respectively. By managing our energy use, we can reduce the cost of electricity by the hour and ultimately by the day. In contrast to unplanned energy consumption patterns, Fig. 14 hourly power cost clearly illustrates that it is lowest during ON-peak hours when our proposed strategy is used. Figure 15 displays the overall cost of daily electricity consumption. When LMMRFO is compared to MRFO and unscheduled electricity consumption, the results show that the cost of electricity is at its lowest. However, the cost of electricity paid with MRFO is less than the cost of unscheduled electricity use. In instance 2, employing MRFO and LMMRFO, the overall electricity cost is reduced by 30% and 40%, respectively.

### Scenario 3: Home with microgrid and energy management

**Figure 18:**
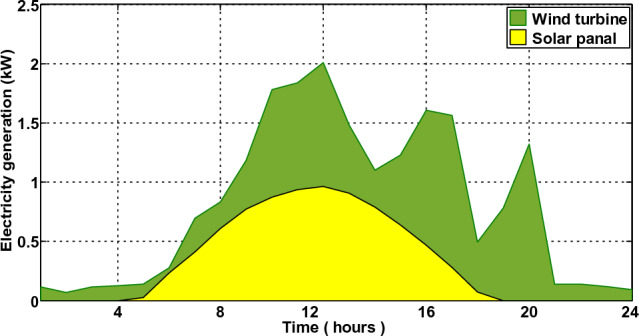
Electricity is generated using solar panels and wind turbines.

**Figure 19:**
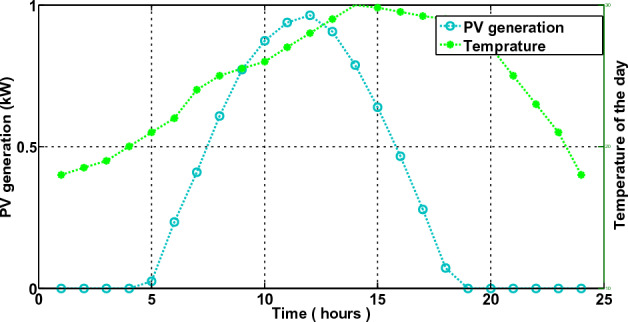
Temperature and the amount of electricity generated by solar panels.

**Figure 20:**
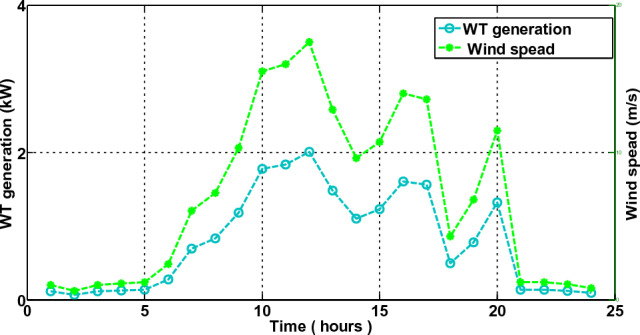
Electricity generated by wind turbines.

**Figure 21:**
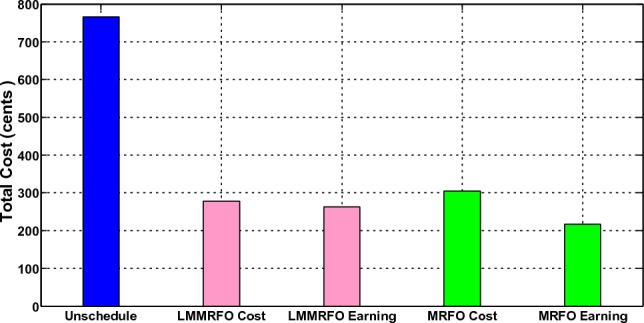
Costs and profits from selling and importing electricity.

**Figure 22:**
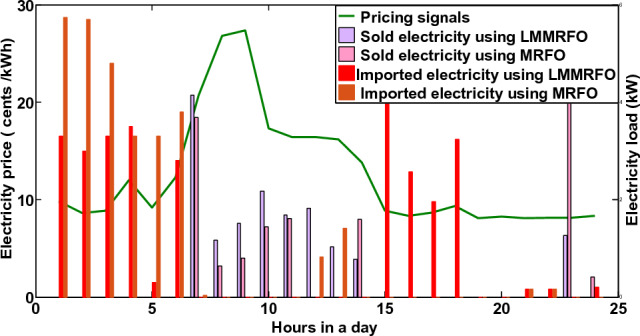
Electricity that is imported and sold with price signals.

In the third scenario, the smart house receives electricity if costs are low, the microgrid meets the load requirement during ON-peak hours, and surplus electricity is sent back to the industrial grid at a loss to the commercial grid. The smart house in this scenario is like to scenario 2, but it includes an ESS with microgrid. Every hour, the smart home can decide whether to switch the load, buy, sell, or store electricity. This action generates the highest profit for the smart home. The electricity produced by a solar panel and wind turbine is depicted in Fig. 18. Because solar irradiation and the wind speed are at their highest during the daytime, the microgrid generates the most electricity during ON-peak times. However, when wind speed and sun irradiation are at their lowest or zero during the evening or early morning, the amount of electricity produced is low. The correlation between electricity produced by solar panels and temperature and electricity produced by wind turbines is shown in Figs. 19 and 20, respectively. Electricity generation is at its peak when wind speed is at its peak, and vice versa. In order to sell the electricity generated and stored during ON-peak hours, load must first be moved during Off-peak hours. A residence that executes this task effectively makes the highest profit potential from power trading. Furthermore, when it comes to moving loads and selling power during high-priced hours, our suggested LMMRFO outperforms MRFO. The overall cost of power compared to imported electricity and the total revenue compared to sell electricity are shown in Fig. 21. Figure 22 displays the total amount of electricity that was imported and sold using the MRFO and LMMRFO algorithms. The outcomes reveal that both algorithms perform well. In comparison to MRFO, our suggested algorithm LMMRFO outperforms it in terms of lowering electricity costs and increasing profits. The main justification for LMMRFO’s effectiveness is that there are fewer parameters that need to be adjusted.

### Performance evaluation

In this section, we compare the three scenarios studies that were previously presented together with our suggested plan that combines MRFO and LMMRFO. Figure 15 shows that the suggested plan employing MRFO and LMMRFO reduced the overall cost of power compared to scenario 1. Figure 17 compares scenario 1 and scenario 2, and the data make it abundantly evident that scenario 2 has a lower PAR than scenario 1. In addition, when we contrast scenarios 2 and 3, as depicted in Fig. 21, in scenario 3, the electricity cost is significantly reduced. Scenario 3 minimizes the total imported electricity from the external grid compared to scenarios 1 and 2, as shown in Fig. 21. For dependable and steady grid functioning in scenario 3, ESS is also taken into consideration. However, power trading is only feasible in scenario 3, and Fig. 21 shows the entire amount of money made via power trading. In Scenario 1, where no specific technique was utilized, the cost amounted to 766 cents, and the Peak to Average Ratio registered at 3.99. Nevertheless, it's important to note that cost savings and earnings were not applicable in this particular scenario. Moving on to Scenario 2, in which the LMMRFO technique was implemented, a notable reduction in cost was observed, with the figure decreasing to 461 cents. Additionally, the Peak to Average Ratio improved significantly, reaching 2.04. This resulted in a cost savings of 40%. However, it's worth mentioning that earnings were not recorded in this case. Conversely, when the MRFO technique was employed, the cost in this scenario amounted to 538 cents, and the Peak to Average Ratio was 3.41. In this context, cost savings of 30% were achieved.

In Scenario 3, we deployed both the LMMRFO and MRFO approaches to efficiently oversee household energy consumption. When utilizing the LMMRFO method, the overall electricity expenses were significantly reduced to 277 cents, marking a remarkable 64% reduction compared to Scenario 1. The Peak to Average Ratio (PAR) saw a notable decrease to 1.78, indicating effective load management. Additionally, this optimization strategy yielded substantial earnings of 263 cents, highlighting its ability to maximize profits through electricity trading.

In contrast, the MRFO technique in Scenario 3 resulted in a slightly higher electricity cost of 310 cents, with a PAR of 2.99. Despite achieving a somewhat lower cost savings of 60% compared to LMMRFO, MRFO still showcased significant enhancements over Scenario 1. Earnings in this scenario amounted to 199 cents, emphasizing its effectiveness in reducing costs and maximizing profits when compared to the baseline scenario. These findings underscore the importance of selecting the appropriate optimization technique, with LMMRFO clearly outperforming MRFO, leading to substantial cost reductions and increased earnings in residential energy management.

One of the most important factors contributing to the improved efficiency, cost-effectiveness, and performance is the implementation of an optimized load management and scheduling algorithm (LMMRFO). This algorithm effectively shifts electricity consumption from peak hours to periods with lower costs, as shown in Fig. 16. In addition, the integration of renewable energy sources, such as solar and wind, enables users to generate electricity and even sell excess energy back to the grid. This not only enhances profits but also reduces electricity expenses, as shown in Figs. 21 and 22. Strategic management of energy use, especially the avoidance of peak hours, has played a crucial role in reducing electricity costs and relieving pressure on the electricity grid.

Table 7 shows the comparison as a whole and the approach presented in^29^. In the Table 7, we compare the results of different scenarios using various techniques, including LMMRFO, MRFO, SA and SCA^29^. It is evident that the improved algorithm (LMMRFO) outperforms the original algorithm in terms of cost and efficiency. This is particularly noticeable in the cost savings percentage, where the enhanced algorithm showcases significant improvements compared to the original. When compared to the results obtained from the reference^29^, the superiority of the improved algorithm becomes even more apparent. The cost savings and earnings demonstrate the effectiveness of the enhanced approach in achieving cost-efficiency and overall better performance compared to the baseline and the reference^29^.

**Table 7:** Comparison between case studies with the approach presented in^29^.

Parameters	Scenario 1	Scenario 2				Scenario 3			
Technique	–	LMMRFO	MRFO	SA^29^	SCA^29^	LMMRFO	MRFO	SA^29^	SCA^29^
Cost (cents)	766	461	538	562.02	487.43	277	310	335.74	284.70
PAR	3.99	2.04	3.41	3.63	2.29	1.78	2.99	3.12	1.98
Cost savings%	–	40	30	26.63	36.42	64	60	56.26	62.83
Earnings (cents)	–	–	–	–	–	263	199	173.39	249.39

The electricity cost in Scenario 2, achieved a substantial cost reduction of 40% with the utilization of LMMRFO, surpassing the MRFO (30%) and SCA (36.42%) approaches. Additionally, the PAR values of 2.04 and 2.29 were obtained using the LMMRFO and SCA techniques, respectively. These outcomes underscore the superior performance of the LMMRFO algorithm in terms of cost savings compared to MRFO and SCA, as well as its efficiency in enhancing PAR.

In Scenario 3, the LMMRFO approach continues to excel, both in terms of electricity cost and PAR.

When focusing on earnings, LMMRFO achieves remarkable results with a earning of 263 cents, while SCA lags behind at 249.39 cents. This emphasizes the cost-efficiency of LMMRFO over SCA. Furthermore, the PAR values further underscore the superiority of LMMRFO, with a PAR of 1.78 compared to SCA's 1.98. These findings demonstrate the consistent performance improvement of LMMRFO across various scenarios, making it a compelling choice for optimizing electricity cost and overall system efficiency.

## Conclusions

A smart home is equipped with various appliances and a microgrid powered by renewable energy sources (RES) to generate electricity. Demand response (DR) technology has become increasingly important in balancing power supply and demand, especially with the development of smart grid technologies. This study proposes a DR scheme based on real-time pricing (RTP) tariffs in residential areas, with the goal of reducing the peak to average ratio (PAR) and electricity costs. By utilizing the manta ray foraging optimization (MRFO) and long-term memory MRFO (LMMRFO) algorithms, as well as an RTP tariff for power trading between users and the commercial grid, an electricity load management plan is created. The plan takes into account both power trading and load scheduling issues in a smart home with a microgrid connected to the grid. To improve the efficiency and reliability of the microgrid, an energy storage system (ESS) is also included. The smart home autonomously decides whether to sell, buy, or store electricity based on pricing and electricity generation signals. Simulation results demonstrate that the proposed strategy outperforms other methods in terms of reducing PAR and power costs while maximizing revenue. Specifically, the proposed plan using MRFO and LMMRFO reduced power costs by 30% and 40% in case 2 and 60% and 64% in case 3, respectively. The earnings from the proposed strategy using MRFO and LMMRFO were 199 and 263 cents, respectively. Overall, the results indicate that LMMRFO performs better than MRFO.

## Data Availability

All data generated or analysed during this study are included in this published article.

## List of symbols

τ: Waiting time
$\eta$: Startup time of each device
$\beta$: Less time to finish each device
$\alpha$: Earliest time to start each device
${E}_{b}$: Total amount of electricity of base device
${\lambda }_{b}$: Power rating of base device
${E}_{ni}$: Total amount of electricity of non-interruptible device
${\lambda }_{ni}$: Power rating of base device of non-interruptible device
${E}_{s}$: Total amount of electricity of shiftable device
${\lambda }_{s}$: Power rating of base device of shiftable device
${\alpha }_{b}$: OFF/ON status of base device
${\alpha }_{ni}$: OFF/ON status of non-interruptible device
${\alpha }_{s}$: OFF/ON status of shiftable device
${\sigma }_{{d}_{b}}^{t}$: Hourly cost against utilized electricity of base device
${\delta }_{{d}_{b}}^{\text{Total}}$: Daily cost against utilized electricity of base device
${\sigma }_{{d}_{ni}}^{t}$: Hourly cost against utilized electricity of non-interruptible device
${\delta }_{{d}_{ni}}^{\text{Total}}$: Daily cost against utilized electricity of non-interruptible device
${\sigma }_{{D}_{s}}^{t}$: Hourly cost against utilized electricity of shiftable device
${\delta }_{{D}_{\mathrm{s}}}^{\text{Total}}$: Daily cost against utilized electricity of shiftable device
$t$: Single time slot
$T$: Maximum time slot
$${D}_{n}$$: All devices
$${D}_{s}$$: Shiftable devices
${D}_{ni}$: Non-interruptible devices
$${D}_{b}$$: Base devices
${d}_{b}$: One device from Base group
${d}_{ni}$: One device from non-interruptible group
${d}_{s}$: One device from shiftable group
$${i}_{L}$$: Light current
${P}_{t}^{wt}$: Wind turbine power
${i}_{S}$: Diode saturation current
$p$: Solar panel power generation
$${R}_{Sh}$$: Shunt resistance
$${R}_{S}$$: Series resistance
${n}_{s}$: Number of solar cells
${\eta }^{ESS}$: The efficiency of $EES$
$E{S}^{ch}$: Charging of ESS
$E{S}^{dis}$: Discharging of ESS
$$SE$$: Stored electricity
$${BC}^{buy}$$: Buying electricity
$${BC}^{sell}$$: Selling electricity
${\varsigma }^{total}$: The overall cost incurred for the imported electricity throughout a day
${\varsigma }^{t}$: The cost of imported electricity on an hourly basis.
${\eta }^{t}$: Total cost of electricity sold
${\k{e} }^{earn}$: The earnings on an hourly basis.
${\eta }^{sell}$: Hourly sold electricity
${\k{e} }^{t}$: The overall earnings for a day
